# Sex differences in delivery and neonatal characteristics of new-borns from the “MAMI-MED” cohort

**DOI:** 10.3389/fpubh.2025.1498125

**Published:** 2025-01-27

**Authors:** Roberta Magnano San Lio, Martina Barchitta, Andrea Maugeri, Elisabetta Campisi, Giuliana Favara, Claudia Ojeda Granados, Claudia La Mastra, Maria Clara La Rosa, Fabiola Galvani, Elisa Pappalardo, Carla Ettore, Giuseppe Ettore, Antonella Agodi

**Affiliations:** ^1^Department of Medical and Surgical Sciences and Advanced Technologies "GF Ingrassia", University of Catania, Catania, Italy; ^2^Department of Obstetrics and Gynaecology, Azienda di Rilievo Nazionale e di Alta Specializzazione (ARNAS) Garibaldi Nesima, Catania, Italy

**Keywords:** pregnancy, sex differences, neonatal outcomes, birth weight, weight for gestational age, maternal health

## Abstract

**Introduction:**

Exploring modifiable and non-modifiable determinants—like sex of new-borns, maternal characteristics, and lifestyle—of maternal and child health is a priority in Public Health. Understanding these sex-based differences is essential for tailored care and informed public health policies.

**Methods:**

The present study aimed to delineate sex disparities in delivery and neonatal characteristics within the “MAMI MED” cohort, an ongoing prospective study involving mother–child pairs from Catania, Italy. The analysis included 1,090 mother–child pairs.

**Results:**

The comparison of birth weight and birth length distribution showed some differences between sexes, confirmed by higher birth weight (*β* = 0.121; 95% CI = 0.071–0.172) and greater birth length (*β* = 0.659; 95% CI = 0.360–0.958) in males compared to females. However, the comparison of small and adequate for gestational age (SGA vs. AGA) revealed that the likelihood of being SGA was higher in males than in females (OR = 1.592; 95% CI = 1.005–2.563).

**Discussion:**

Thus, the focus should be on improving the assessment of gender-based differences in diagnostic criteria and incorporating gender-specific aspects into existing preventive guidelines to deeply understand the effect of gender disparities and risk factors on maternal-child health.

## 1 Introduction

In line with the principles of Predictive, Preventive, Personalized, and Participatory (P4) Medicine, it is imperative to harmonize various dimensions of biological data, encompassing molecular, cellular, and phenotypical assessments, alongside the individual genome sequences. This integration is vital for safeguarding health and enhancing well-being across all stages of life (1, 2). In this context, both biological and socio-cultural factors can influence various aspects, including risk factors, prevalence, age of onset, clinical presentation, prognosis, biomarkers, and treatment effectiveness (3). Notably, evidence reveals gender disparities in chronic conditions like diabetes, cardiovascular diseases, neurological disorders (4), cancer (5), and aging (6). Furthermore, differences in lifestyles such as diet, physical activity, tobacco and alcohol consumption play a role in the epidemiology of diseases (7). Therefore, the challenge lies in translating research excellence into clinical practice, customized to an individual’s genetic profile, lifestyle, and environment (8). Men and women, especially during their reproductive years, encounter health determinants in distinct ways. The World Health Organization introduced the Social Determinants of Health in 2008 to identify and address health disparities across different population groups (9). It is important to acknowledge that women’s health during their reproductive years significantly influences their long-term well-being and that of their families. In this context, the Developmental Origins of Health and Disease (DOHaD) hypothesis posits that the uterine environment programs the fetus to cope with challenges it is likely to face after birth (10). In particular, the initial one thousand days of life, spanning from conception to the conclusion of the second year, constitute the primary critical window that establishes the groundwork for lifelong development and well-being (11). Exploring the key determinants, both modifiable and non-modifiable, that influence maternal and child health is a top priority in the field of Public Health. Factors known to directly influence intrauterine growth encompass infant sex, racial or ethnic background, maternal height, pre-pregnancy weight, maternal birth weight, parity, gestational weight gain, caloric intake, and cigarette smoking (12). Of particular interest among non-modifiable factors are the sex disparities in childbirth and neonatal attributes, which have long captivated the attention of researchers and healthcare experts (13). This field of research examines potential variations in crucial outcomes between male and female new-borns. Male and female foetuses respond differently to the same intrauterine environment, suggesting a fundamental biological difference at the cellular and molecular level. Understanding these sex-based distinctions is pivotal in the realm of perinatal care, as they can have far-reaching implications for the health and well-being of new-borns. Nonetheless, there is a scarcity of studies investigating the influence of sex on neonatal and child health, and the findings obtained so far exhibit a diversity of outcomes. While it is broadly recognized that males are more susceptible to several adverse pregnancy outcomes and complications (14), this male disadvantage is not consistently evident across all pregnancy-related issues or throughout the entire gestational period.

Due to a wide range of risk factors, male new-borns may have slightly higher rates of neonatal mortality compared to female new-borns (15–17). Infant males face a higher risk of respiratory and gastrointestinal infections, which may be attributed to elevated testosterone levels that can suppress the immune system (18, 19). Sex differences could be also important in the long-life health, influencing different cognitive abilities between male and female new-borns in the early years of life (20). Additionally, the outcomes of interest include anthropometric measurements (i.e., birth weight and length, and assessments of weight in relation to gestational age), gestational age at delivery, and preterm birth rates. In particular, birth weight and length are fundamental indicators of a baby’s growth and development, while gestational age at delivery and preterm birth are crucial determinants of neonatal health.

Our study goes beyond traditional metrics of birth weight and gestational age by incorporating a comprehensive range of maternal factors and evaluating birth weight in relation to gestational age. By analyzing these data, we aim to uncover trends and disparities between male and female newborns. While it is well-documented that gender differences exist in fetal and postnatal developmental trajectories, and that the charts used to define appropriate or inadequate weight for gestational age are differentiated by gender, there remains a significant gap in understanding whether gender itself influences the risk of adverse outcomes, such as inadequate birth weight for gestational age. To date, the literature has not conclusively established how gender affects these risks. Using data from the “MAMI MED” cohort, our study seeks to address this gap by examining whether being male or female impacts the likelihood of adverse neonatal outcomes. This cohort offers a unique perspective on sex-specific trends within a Mediterranean population, which may differ from those observed in other, more extensively studied populations due to variations in social, environmental, and genetic factors. By focusing on this Mediterranean context, our research provides valuable insights into global discussions on neonatal health and development. In particular, the present analysis offers an examination of sex differences in delivery and neonatal characteristics within the “MAMI MED” cohort. This information is crucial for informing clinical practices, healthcare interventions, and public health policies designed to ensure the best possible start for every infant.

## 2 Materials and methods

### 2.1 Study population

**Figure 1:**
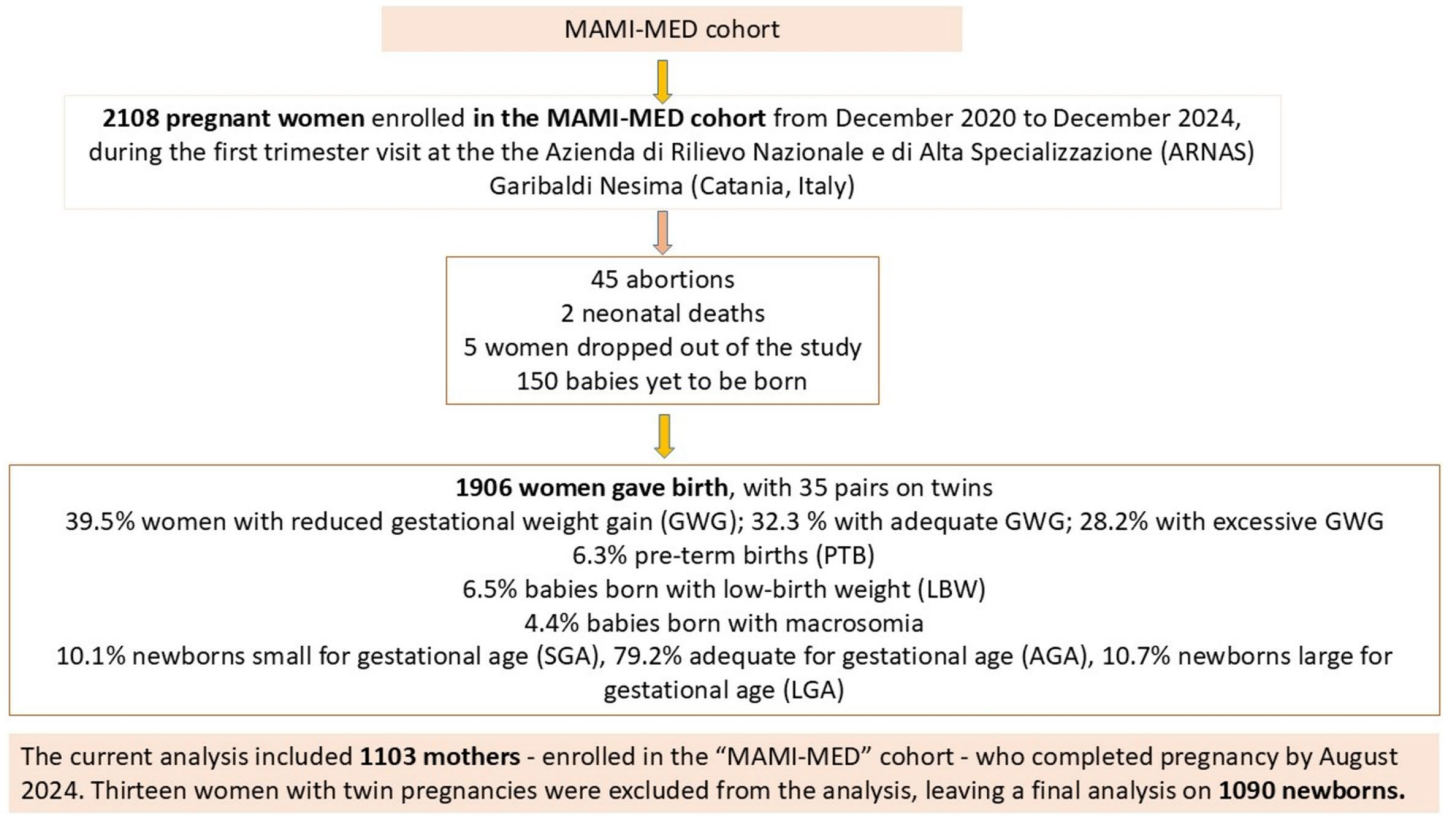
Flowchart of MAMI-MED cohort.

The present analysis is based on data from the “MAMI-MED” cohort, an ongoing prospective study involving mother–child pairs from Catania, Italy, established in December 2020. The general goal of this study is to assess how social, environmental, behavioural, and molecular factors impact the health of both mothers and children. The “MAMI-MED” cohort follows the same study protocol and methodologies as the “Mamma and Bambino” cohort, which was established in 2015 (21–25). Briefly, the study population comprises pregnant women who are enrolled during their first-trimester visit at the Azienda di Rilievo Nazionale e di Alta Specializzazione (ARNAS) Garibaldi Nesima in Catania, Italy, where they undergo the bi-test screening. In this context, women included in the study are interviewed for the first time. Detailed information about antenatal visits and pregnancy protocols followed prior to and during pregnancy is not collected, except for data gathered during the follow-up at delivery. Specifically, women are fully informed about the study’s objectives and provide their informed consent before being included in the study. Subsequently, two tailored questionnaires are administered to the women. The first questionnaire investigates sociodemographic factors such as educational level and employment, smoking habits, diagnosed pathologies, pre-pregnancy height and weight, and the use of folic acid and other supplements. The second questionnaire focuses on the woman’s dietary habits, collected using the Food Frequency Questionnaire (FFQ). The research plan also involves follow-up interviews, conducted at delivery, and then at 12, 24, and 48 months postpartum, to gather data on maternal and child health. The delivery questionnaire gathers information on gestational duration, type of delivery, baby’s birth weight and length, woman’s weight at delivery, and any complications during pregnancy, including gestational diabetes mellitus, antibiotic use during or after pregnancy, and vaccinations received by the mother. Data collected during the subsequent follow-ups primarily concern the child’s growth, health, breastfeeding, and weaning, as well as daily habits such as sleep quality, time spent outdoors and in front of electronic devices, and time spent with pets. Additionally, factors influencing pollution are analyzed, including residential traffic levels, the floor of the residence, and parental smoking. This study adheres to the principles outlined in the Declaration of Helsinki and received approval from the Ethics Committee “Catania 2” under protocol number Prot. N. 487/CE, 71/2020/CECT2; Prot. N. 762/CE, 83/2021/CECT2; Prot. N. 108/CE, 100/CECT2. All participating women provide an informed consent after being thoroughly briefed on the study’s objectives. For the present analysis, we included mother–child pairs with complete datasets covering socio-demographic information, delivery characteristics, and neonatal outcomes. Women with pre-existing conditions, such as hypertension, diabetes, or autoimmune disorders, as well as those with pregnancy complications like preeclampsia or gestational diabetes, were excluded from the analysis. Additionally, only women who deliver at the ARNAS Garibaldi Nesima hospital were included in the study. Similarly, mother–child pairs with multiple pregnancies or congenital diseases were also excluded, as these factors could affect neonatal outcomes and potentially bias the assessment of sex differences. Figure 1 shows details on study population.

### 2.2 Data collection

At the time of enrollment, each woman completed a structured questionnaire aimed at gathering socio-demographic information and behavioral data. Maternal education was categorized into three groups (i.e., low, medium and high), with a low educational level for women with ≤8 years of schooling and a high educational level for those with >8 years of schooling. Maternal employment status was also assessed, distinguishing between unemployed individuals—including students and housewives—and those who were employed. Smoking status was classified as either current smokers or non-current smokers (including ex-smokers). Additionally, women were queried about their height and weight prior to pregnancy at the point of recruitment. This data was subsequently utilized to calculate and categorize pre-pregnancy BMI in accordance with WHO criteria: (i) underweight, with BMI (Kg/m^2^) < 18.5; (ii) normal weight, with BMI (Kg/m^2^) 18.5–24.9; (iii) overweight, with BMI (Kg/m^2^) ≥ 25.0–29.9, and (iv) obese, with BMI (Kg/m^2^) ≥ 30.0 (21). To compute total gestational weight gain (GWG), the self-reported pre-pregnancy weight was subtracted from the weight recorded at the time of delivery. Adequate GWG was defined based on Institute of Medicine guidelines, which specify a weight gain of 12.5 to 18 kg for underweight women, 11.5 to 16 kg for normal-weight women, 7 to 11.5 kg for overweight women, and 5 to 9 kg for obese women (22). Neonatal and delivery characteristics—such as gestational age at delivery, birth weight, and birth length—were also collected. The primary birth outcomes of interest included pre-term birth—PTB, birth weight for gestational age—categorized as small for gestational age, SGA; appropriate for gestational age, AGA, or large for gestational age, LGA—based on sex-specific national reference charts [23], and macrosomia (birth weight > 4 Kg). Specifically, the cut-offs for birth weight for gestational age were as follow: (i) SGA—Birth weight below the 10th percentile for gestational age and sex; (ii) AGA: Birth weight between the 10th and 90th percentiles for gestational age and sex, and (iii) LGA: Birth weight above the 90th percentile for gestational age and sex. These percentiles were derived from the reference charts that account for both the gestational age and sex of the neonate, ensuring accurate categorization tailored to the population studied. At recruitment, the assessment included a 95-item semiquantitative Food Frequency Questionnaire (FFQ) referred to the previous 30 days (24, 25). Based on the information collected from the FFQ, the overall energy consumption was additionally calculated utilizing the US Department of Agriculture (USDA) Food Composition Database,^1^ which was adapted to account for Italian foods. Daily dietary intakes were normalized with respect to total energy intake using the residual method (26).

### 2.3 Statistical analyses

Statistical analyses were carried out utilizing SPSS v. 26.0 (IBM Corp., Armonk, NY, United States). Descriptive statistics, including medians with interquartile ranges (IQR) or frequencies with percentages (%), were employed in univariate analysis to describe the study population’s characteristics. The distribution of quantitative variables was assessed using the Kolmogorov–Smirnov test. For bivariate analysis, the Mann–Whitney U-test was employed for quantitative variables, while the Chi-squared test was used for categorical variables. Multivariable linear regression analyses were conducted to assess the relationship between sex and both birth length and weight, while adjusting for potential confounders, including maternal age, gestational age at delivery, pre-gestational BMI, GWG, total energy intake, smoking status, and delivery method. Specifically, continuous variables included maternal age, gestational age at delivery, pre-gestational BMI, gestational weight gain (GWG), and total energy intake. Binary variables included smoking status (smokers vs. non-smokers/former smokers) and delivery method (caesarean vs. natural). Multivariable logistic regression analyses were conducted to evaluate the association between sex and adverse birth outcomes, specifically PTB and weight for gestational age categories (SGA vs. AGA, and LGA vs. AGA). Results from linear regression models are presented as beta regression coefficients with 95% confidence intervals (95%CIs), while logistic regression results are reported as odds ratios (ORs) with 95%CIs. All statistical tests were two-sided and conducted at a significance level of *α* = 0.05.

## 3 Results

The current analysis included 1,103 mothers—enrolled in the “MAMI-MED” cohort—who completed pregnancy by August 2024. Table 1 describes the main characteristics of women, which reported a median age of 31 years (IQR = 7) and a median gestational age at recruitment of 12 weeks (IQR = 0). With respect to socioeconomic characteristics, 50.3% of women had a medium educational level, while 25.7 and 24.0% of them reported low and high educational level, respectively. Accordingly, 51.4% of women were employed. Based on pre-gestational BMI, we identified 6.0% underweight, 58.3% normal weight, 22.3% overweight and 13.4% obese women. According to pre-gestational BMI and GWG, we considered 39.3 and 27.9% who exhibited reduced or excessive GWG, respectively.

**Table 1:** Characteristics of mothers enrolled in the “MAMI-MED” cohort.

Characteristics (*N* = 1,103)	Median (IQR) or frequency (%)
Age (years)	31.0 (7)
Gestational age at recruitment (weeks)	12.0 (0)
Educational level	
Low	25.7%
Medium	50.3%
High	24.0%
Employed (% yes)	51.4%
Smokers (% yes)	9.7%
Parity (% yes)	47.1%
Pre-gestational Body Mass Index	23.3 (6.0)
BMI-categories	
Underweight	6.0%
Normal weight	58.3%
Overweight	22.3%
Obese	13.4%
Gestational weight gain (GWG)	11.0 (7.0)
GWG categories	
Reduced	39.3%
Adequate	32.8%
Excessive	27.9%
Total energy intake (Kcal)	1691.7 (568.9)

Thirteen women with twin pregnancies were excluded from the analysis, leaving a final dataset of 1,090 newborns (Table 2). Of these, 50.8% were male (*N* = 567) and 49.2% were female (*N* = 536). Regarding delivery details, the median gestational age at birth was 39.0 weeks (IQR = 2), with 5.9% of births occurring pre-term. Caesarean sections accounted for 28.2% of all deliveries. In terms of neonatal characteristics, the median birth weight was 3.3 kg (IQR = 0.5), and the median birth length was 50.0 cm (IQR = 2). Macrosomia was observed in 4.0% of newborns. Notably, 80.5% of newborns were classified as AGA, while 8.6% SGA and 10.9% were LGA.

**Table 2:** Sex differences in delivery and neonatal characteristics.

Characteristics	Median (IQR) or frequency (%)			*p*-value^a^
	Overall (*N* = 1,090)	Males (*N* = 567)	Female (*N* = 536)	
Gestational age at delivery (weeks)	39 (2)	39 (2)	39 (2)	0.785
Caesarean section	28.2%	28.0%	28.5%	0.874
Preterm birth	5.9%	5.6%	6.2%	0.676
Birth weight (kg)	3.3 (0.5)	3.4 (0.6)	3.2 (0.5)	<0.001
Birth length (cm)	50.0 (2.0)	50.0 (3.0)	50.0 (3.0)	<0.001
Macrosomia	4.0%	5.6%	2.3%	0.005
Small-for-gestational age	8.6%	10.5%	6.7%	0.027
Large-for-gestational age	10.9%	10.7%	11.0%	0.972

a Results are based on the Mann–Whitney U-test for quantitative variables and the Chi-squared test for categorical variables.

When stratified by sex, birth weight distribution showed significant differences, with males having a higher median birth weight (3.4 kg; IQR = 0.6) compared to females (3.2 kg; IQR = 0.6; *p* < 0.001). Additionally, macrosomia was more common among male newborns than females (5.6% vs. 2.3%; *p* = 0.005). While the difference might not be immediately evident from the median values (50.0 cm for both sexes), the Mann–Whitney test detected a statistically significant difference in the distributions of birth length between males and females (*p* < 0.001). No significant sex differences were observed for gestational age at delivery or PTB. However, when accounting for birth weight relative to gestational age, a higher proportion of male newborns were classified as SGA compared to females (10.5% vs. 6.7%; *p* = 0.027), while the proportion of LGA was similar between the two sexes (10.7 and 11.0%, respectively; *p* = 0.972).

We then conducted regression analyses to evaluate sex differences in neonatal outcomes within the “MAMI MED” cohort. Linear regression results indicated that male newborns had significantly higher birth weights than females (*β* = 0.112; 95% CI = 0.052–0.171; *p* < 0.001). Similarly, males were found to have greater birth lengths compared to females (*β* = 0.583; 95% CI = 0.253–0.913; *p* = 0.001). The positive associations between male sex and both birth weight and length remained significant after adjusting for maternal age, gestational age at delivery, pre-gestational BMI, GWG, total energy intake, smoking status, and delivery method. Males continued to show higher birth weight (β = 0.121; 95% CI = 0.071–0.172; *p* < 0.001) and greater birth length (β = 0.659; 95% CI = 0.360–0.958; p < 0.001; Table 3). Logistic regression models were used to examine sex differences in the likelihood of macrosomia and inadequate birth weight for gestational age. The probability of macrosomia was approximately 60% lower in males compared to females (OR = 0.388; 95% CI = 0.197–0.764; *p* = 0.006), even after adjusting for maternal age, gestational age at delivery, pre-gestational BMI, GWG, total energy intake, smoking status, and delivery method (OR = 0.443; 95% CI = 0.217–0.904; *p* = 0.025). In contrast, males were 1.6 times more likely than females to be classified as SGA compared to AGA (OR = 1.640; 95% CI = 1.055–2.550; *p* = 0.028), a result that remained significant after adjusting for potential confounders (OR = 1.592; 95% CI = 1.005–2.563; *p* = 0.045; Table 4). No significant association was found with gestational age at delivery, PTB, or LGA.

**Table 3:** Linear regression models to evaluate sex differences in neonatal outcomes within the “MAMI MED” cohort.

Characteristics	Model	β	95%CI	*p*-value
Birth weight	Unadjusted	0.112	0.052–0.171	<0.001
	Adjusted	0.121	0.071–0.172	<0.001
Birth length	Unadjusted	0.583	0.253–0.91	0.001
	Adjusted	0.659	0.360–0.958	<0.001

The adjusted model accounts for maternal age, gestational age at delivery, pre-gestational BMI, gestational weight gain (GWG), total energy intake, smoking status, and delivery method. Results are presented as beta coefficients with 95% Confidence Intervals (CIs) for the association between male sex and neonatal outcomes.

**Table 4:** Logistic regression models to evaluate sex differences in neonatal outcomes within the “MAMI MED” cohort.

Characteristics	Model	OR	95%CI	*p*-value
Macrosomia	Unadjusted	0.388	0.197–0.764	0.006
	Adjusted	0.443	0.217–0.904	0.025
SGA vs. AGA	Unadjusted	1.640	1.055–2.550	0.028
	Adjusted	1.592	1.005–2.563	0.045

The adjusted model accounts for maternal age, gestational age at delivery, pre-gestational BMI, gestational weight gain (GWG), total energy intake, smoking status, and delivery method. Results are presented as Odds Ratios (ORs) with 95% Confidence Intervals (CIs) for the association between male sex and neonatal outcomes.

## 4 Discussion and conclusion

The present analysis outlines variations in delivery and neonatal outcomes among male and female new-borns within the “MAMI MED” cohort, while considering various potential factors that might confound the sex-related effects.

Our findings align with previous comparisons showing that males tend to be larger starting from the second trimester of pregnancy (27, 28), with higher birth weight compared to females (12, 29, 30). Specifically, our comparison of birth weight distribution showed some differences between sexes, with higher values for males compared to females. Linear regression models confirmed that males have a greater birth weight than females, even after accounting for potential confounding variables such as maternal age, gestational age at delivery, pre-gestational BMI, GWG, and total energy intake. Similarly, the comparison of birth length distribution showed significant differences between sexes, and linear regression model confirmed that male new-borns have higher birth length compared to females.

Although females have lower birth weight and length than males, the underlying reasons have not been thoroughly investigated (31, 32). One possible explanation may lie in the fact that birth anthropometric measurements are significantly impacted by the gestational week at delivery, with possible sex disparities in this regard. In fact, sex may also influence gestational age at birth and the risk of Pre-term birth (PTB), one of the major cause of death among newborns and the second leading cause of death under 5 years (33). Preterm new-borns require specialized care and closer monitoring to address potential complications. Although PTB is common, its etiology is still unclear. One of the prominent risk factors is that male infants may have a greater propensity for preterm birth compared to their female counterparts (34). Research in Western nations has revealed a connection between women carrying male foetuses and an elevated risk of PTB (35–38). These findings have been substantiated by recent investigations in non-Western regions as well (39, 40). Consequently, male foetuses might be considered an independent risk factor for spontaneous PTB, regardless of other contributing factors (16, 34, 35, 38, 41, 42). Additionally, when examining data based on gestational age, it has been noted that the male predominance in cases of spontaneous PTB is more pronounced in the earlier stages of gestation (34, 38, 41). Despite this evidence, our analysis did not reveal any significant difference in gestational age at delivery and pre-term births between sexes. Despite the evidence in the literature suggesting a higher propensity for preterm birth in male infants, our results did not show significant differences in gestational age at delivery or in the incidence of preterm births between the sexes. It is important to consider that our cohort has a relatively low overall rate of PTB (5.9%), which may limit the statistical power needed to detect significant differences in this outcome. Additionally, the cohort studied has specific characteristics, such as a relatively young median maternal age and a variety of socioeconomic factors and BMI categories, which may influence our findings. Additionally, the heterogeneity of results may be attributed to potential variations that hinge on the clinical causes of preterm birth and other associated risk factors, including pre-existing comorbidities, genetic and environmental risks that can differ among populations. In fact, women with pre-existing conditions, such as hypertension, diabetes, or autoimmune disorders, as well as those with pregnancy complications, including preeclampsia and gestational diabetes, were excluded from our analysis. These groups are known to have a higher risk of PTB, and their omission may have significantly impacted the observed results. Therefore, while the literature suggests a higher prevalence of PTB in male fetuses, further research in larger cohorts or longitudinal studies that account for different maternal characteristics and environmental exposures could provide more insights into the sex-specific mechanisms influencing PTB and help explain the variability of findings across studies.

Revisiting anthropometric measurements at birth, our analysis specifically included the assessment of birth weight in relation to gestational age. In particular, the rate of SGA infants is defined as the proportion of live births whose birth weight falls below the standard 10th percentile of birth weight for gestational age, taking into account the infant’s sex, within a specific location and time frame. In some cases, different thresholds, like the 3rd percentile of birth weight for gestational age, have also been employed to identify SGA. It’s important to note that SGA and intrauterine growth restriction (IUGR) are often used interchangeably, but they do have distinct differences. IUGR refers to poor fetal growth, which can result from various mechanisms, while SGA describes an infant’s position on growth charts after birth without considering the cause for small size or the growth trajectory in utero. In the absence of a diagnostic standard, a variety of metrics—including fetal biometry, Doppler ultrasound, and SGA—are used across studies to define fetal growth restriction. In the present study, we considered the classification of weight for gestational age based on sex-specific national reference charts [23]. While our analysis did not uncover sex disparities in weight for gestational age overall, a more focused examination comparing SGA and AGA infants revealed that the likelihood of being SGA was nearly 1.6 times higher in males than in females. This remained evident even after accounting for potential confounding variables. Given this, it is crucial to highlight that the use of growth curves that do not differentiate by sex may lead to potential overdiagnosis of SGA in females. However, in studies that employ sex-specific growth curves, SGA females seem to have a reduced likelihood of encountering adverse outcomes compared to SGA males (43).

From a biological perspective, sex differences in both healthy and complicated pregnancies are due to the actions of sex chromosomes and steroid hormones, which rise to molecular effects (e.g., sex-specific gene expression patterns, variations in crucial pregnancy hormones and distinctions in the fetoplacental reaction to maternal inflammation and infection) (44, 45). For instance, the heightened pro-inflammatory response to infection in the trophoblast of pregnancies with male foetuses could potentially contribute to the increased occurrence of early spontaneous PTB. Male foetuses display a more pro-inflammatory immune response throughout gestation and face an increased risk of infection, which can result in pregnancy complications (41, 46, 47). These factors may, in turn, play a role in the sex-based variations in early susceptibility to other childhood conditions.

The present study uniquely investigates the variations in delivery and neonatal outcomes between male and female new-borns. By examining potential confounding factors such as maternal age, pre-gestational BMI, and gestational age at delivery, the analysis provides a nuanced understanding of sex-related disparities, particularly in birth weight and length. The findings corroborate prior research showing that male new-borns tend to have higher birth weights and lengths compared to females. These consistent results enhance the study’s generalizability and validate its methodology against established evidence. Additionally, the comprehensive adjustment for potential confounding variables enhances the reliability of the findings. The study’s analysis of SGA highlights the importance of employing sex-specific growth curves to avoid potential overdiagnosis of SGA in female infants, contributing to a deeper understanding of growth-related disparities. The study underscores the importance of addressing sex-specific disparities in neonatal outcomes to inform precision medicine and public health strategies. Its findings advocate for the development of gender-informed diagnostic criteria and preventive measures, contributing to improved maternal and child health outcomes. These strengths collectively position the study as a significant contribution to the understanding of sex-based differences in neonatal outcomes, with implications for research, clinical practice, and public health.

However, our study has several limitations that warrant careful consideration. First, the low occurrence rates of adverse outcomes may have affected the statistical power of our analyses, limiting the ability to detect significant associations in some cases. A significant factor contributing to the low PTB rate in our cohort could be the exclusion criteria applied during participant selection, regarding women with pre-existing conditions, such as hypertension, diabetes, or autoimmune disorders, and with pregnancy complications, such as preeclampsia and gestational diabetes. Since these women have normally a higher risk of PTB, the discrepancy between our findings and literature reporting a higher prevalence of PTB in male foetuses could be partially explained by these exclusion criteria. Additionally, we were unable to analyze asymmetric and symmetric growth patterns in SGA infants due to the lack of data on intrauterine growth trajectories. While existing literature suggests that these growth patterns may vary by gender, our data is limited to neonatal measurements at birth, preventing us from evaluating how gender may influence intrauterine growth dynamics. Future studies that incorporate longitudinal intrauterine growth data would provide a more comprehensive understanding of this aspect. Another notable limitation is the absence of information on maternal physical activity levels before and during pregnancy. Given the evidence that maternal physical activity can impact neonatal outcomes, including birth weight and overall growth, this missing variable leaves a gap in our ability to assess its influence on the observed gender differences in neonatal anthropometric measures. Future research should address this gap by incorporating data on physical activity, which may provide important insights into the factors affecting neonatal growth. Additionally, the study relies on self-reported data for maternal characteristics collected at recruitment and delivery, including dietary habits obtained through FFQ and neonatal anthropometric measurements. Self-reported data are inherently prone to recall and reporting biases, which may have introduced some inaccuracies into our findings. Furthermore, variability in the diagnostic criteria used to classify pregnancy complications across different institutions poses another limitation. These inconsistencies may influence the assessment of birth weight relative to gestational age, and could result in biases when categorizing neonates as SGA or LGA. Lastly, while sex-specific national reference charts are commonly used in neonatal research, discrepancies across studies highlight the need to standardize the criteria for determining neonatal outcomes such as SGA and LGA. A more uniform approach would help to ensure more consistent and reliable comparisons in future research (48).

In this context, also exploring the long-term implications of neonatal outcomes on child health, particularly in areas such as cognitive development and disease susceptibility, is essential for a comprehensive understanding. Research has shown that neonatal factors, including birth weight and gestational age, can significantly influence long-term outcomes. For instance, low birth weight has been linked to cognitive delays, learning disabilities, and an increased risk of metabolic disorders in later life (49). Similarly, preterm birth has been associated with developmental delays, particularly in language and motor skills. Specifically, preterm males exhibited lower cognitive performance and greater motor impairments compared to their female counterparts, potentially due to variations in white matter development. The link between severe brain injury, early pain experiences, and neurodevelopmental outcomes was influenced by sex, suggesting that males and females born preterm respond differently to early-life adversity (50, 51). While our study primarily focused on immediate neonatal characteristics and sex differences, we acknowledge that the impact of these factors on later health outcomes warrants further investigation. Studies have also shown that sex differences in early life may influence vulnerability to diseases such as cardiovascular conditions and obesity (52–54). Future research could build upon this analysis by incorporating longitudinal data to examine the potential long-term effects on cognitive and physical development.

Notwithstanding these limitations, the study revealed that there are significant sex-based differences in neonatal outcomes within the “MAMI MED” cohort, particularly in terms of birth weight and length. Males generally exhibited higher birth weight and length compared to females, a trend that remained consistent even after adjusting for various potential confounding factors. Additionally, males were more likely to be classified as SGA, while the likelihood of macrosomia was lower in males than in females. These results highlight the importance of considering sex-specific factors in neonatal assessments and underscore the need for sex-tailored diagnostic and therapeutic strategies in clinical practice. While the study did not find significant differences in other outcomes such as gestational age at delivery or pre-term births, the observed differences in birth weight and length suggest that further research is needed to explore the underlying biological mechanisms and their implications for maternal and child health. Thus, our study underscores the significance of delving into the mechanisms underpinning the disparities seen in pre- and perinatal complications between genders. This exploration holds promise for developing more effective gender-informed diagnostic and therapeutic approaches. The initial move, nonetheless, involves enhancing the evaluation of sex disparities in diagnostic criteria and integrating sex-specific factors into the existing preventive guidelines from a precision medicine standpoint. To achieve this, it is crucial to promote additional research for a deeper comprehension of the interplay between sex and gender disparities and various risk factors concerning maternal and child health. This knowledge will, in turn, inform future Public Health strategies designed to safeguard the well-being of both mothers and their children.

## Data Availability

The raw data supporting the conclusions of this article will be made available by the authors, without undue reservation.

## Footnotes

^1^ https://www.fns.usda.gov/usda-fis/usda-foods-database
